# Crosstalk between oxidative stress and epigenetic marks: New roles and therapeutic implications in cardiac fibrosis

**DOI:** 10.1016/j.redox.2023.102820

**Published:** 2023-07-17

**Authors:** Zhi-Yan Liu, Kai Song, Bin Tu, Li-Chan Lin, He Sun, Yang Zhou, Rui Li, Yan Shi, Jing-Jing Yang, Ye Zhang, Jian-Yuan Zhao, Hui Tao

**Affiliations:** a Department of Anesthesiology and Perioperative Medicine, The Second Affiliated Hospital of Anhui Medical University, Hefei, 230601, China; b Department of Cardiothoracic Surgery, The Second Affiliated Hospital of Anhui Medical University, Hefei, 230601, China; c Institute for Developmental and Regenerative Cardiovascular Medicine, MOE-Shanghai Key Laboratory of Children's Environmental Health, Xinhua Hospital, Shanghai Jiao Tong University School of Medicine, Shanghai, 200092, China; d Department of Clinical Pharmacology, The Second Affiliated Hospital of Anhui Medical University, Hefei, 230601, China

**Keywords:** Epigenetic, Cardiac fibrosis, Oxidative stress, Extracellular matrix

## Abstract

With the in-depth investigation of cardiac fibrosis, oxidative stress (OS) has been recognized as a significant pathophysiological pathway involved in cardiac remodeling and progression. OS is a condition characterized by the disruption of equilibrium between reactive oxygen species (ROS) produced by the organism and the antioxidant defense system, resulting in adverse effects on the structure and function of the heart. The accumulation of reactive substances beyond cellular thresholds disrupts the normal physiology of both cardiomyocytes and non-cardiomyocytes, leading to OS, inflammation, hypertrophy, and cardiac fibrosis. Furthermore, cardiac OS also modulates several crucial genes involved in maintaining cellular homeostasis, including those associated with mitochondrial biogenesis, injury, and antioxidant defense, which are inevitably associated with concurrent epigenetic changes. Multiple studies have demonstrated the crucial role of epigenetic modifications in regulating cardiac OS. Consequently, modulating OS through targeted epigenetic modifications emerges as a potentially promising therapeutic strategy for managing cardiac fibrosis. This article provides a new review of current research on this subject and proposes that epigenetics may improve OS-induced cardiac fibrosis.

## Highlights

• Oxidative stress mediated epigenetic regulations of cardiac fibrosis.
• Epigenetic regulations of oxidative stress in cardiac fibrosis.
• Oxidative stress and epigenetic crosstalk play a role in cardiac fibrosis.

## Graphical abstract

**Figure:**
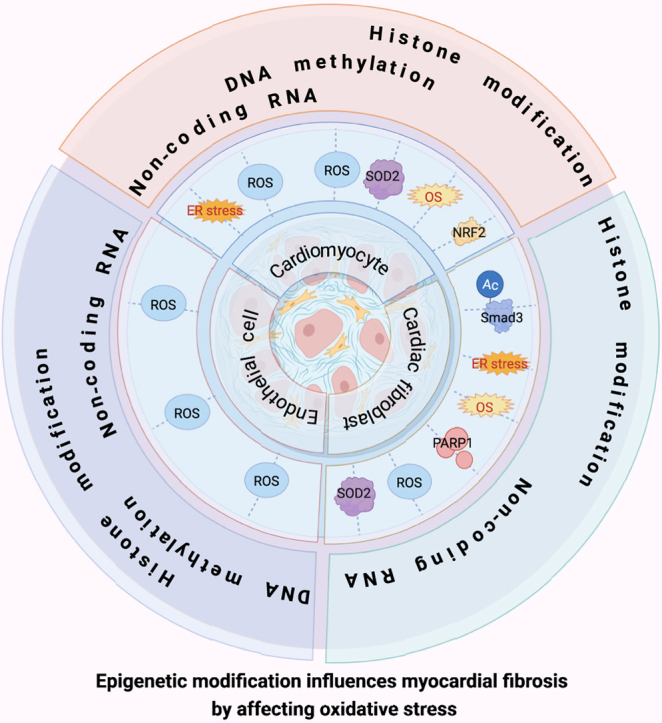

## Abbreviations

KAT7: lysine acetyltransferase 7
LARP7: la-related protein 7
AAV: adeno-associated virus
AngII: angiotensin II
ATM: ataxia-telangiectasia mutated
Bax: Bcl-2-associated X
Bcl-2: B-cell lymphoma-2
CAT: catalase
CHD: coronary heart disease
CircRNAs: circular RNAs
CircITCH: circular RNA itchy E3 ubiquitin protein ligase
CM: cardiomyocyte
CRM1: chromosome region maintenance protein 1
DCM: diabetic cardiomyopathy
DDR: DNA damage response
DNMT: DNA methyltransferase
ECM: extracellular matrix
EC-SOD: extracellular superoxide dismutas
EndMT: endothelial-to-mesenchymal transition
ER: endoplasmic reticulum
ERK1/2: extracellular signal-related kinases 1 and 2
ETC: electron transport chain
EZH2: enhancer of zeste 2 polycomb repressive complex 2 subunit
FOXO1/3: forkhead box protein O1/3
GE: geniposide
GPX1: glutathione peroxidase 1
GSH: glutathione
HAT: histone acetyltransferase
HDACs: histone deacetylases
Hmox-1: heme oxygenase 1
HMTs: histone methyltransferases
HKL: honokiol
IDH: isocitrate dehydrogenase
IGFBP3: insulin-like growth factor-binding protein 3
Imp: importin
IRE1: inositol-requiring enzyme 1
lncRNA: long non-coding RNA
LRP6: lipoprotein receptor-related protein 6
MALAT1: metastasis-associated lung adenocarcinoma transcript 1
Mcl-1: myeloid cell leukemia 1
MDA: malondialdehyde
MIF: macrophage migration inhibitory factor
MMPs: matrix metalloproteinases
Mn-SOD: manganese SOD
mPTP: mitochondrial permeability transition pore
NAD+: nicotinamide adenine dinucleotide
NcRNAs: non-coding RNAs
NEU: neuraminidase
Nfe2l2: nuclear factor (erythroid-derived 2)-like 2
NFIA: nuclear factor I-A
NRF2: nuclear factor-erythroid 2-related factor 2
OGG1: 8-oxoguanine DNA glycosylase
OS: oxidative stress
PAK2: p21-activated kinase 2
PGC-1α: peroxisome proliferator-activated receptor γ coactivator 1α
RASSF1A: ras association domain family 1 isoform A
ROS: reactive oxygen species
Sal B: salvianolic acid B
siRNAs: small interfering RNAs
SOD: superoxide dismutase
TBP-2: thioredoxin-binding protein-2
TET: ten-eleven translocation
TRX1: thioredoxin 1
USP22: ubiquitin-specific peptidase 22
3′-UTR: 3‘-untranslated region
VCPs: valosin containing proteins
VDAC1: voltage-dependent anion channel 1
XBP1: X-box-binding protein 1

## 1 Introduction

Cardiac fibrosis, is a multifactorial process that includes, but is not limited to, cardiac fibroblast activation, apoptosis of cardiomyocytes, transition of endothelial cells to mesenchymal cells, injury or aging of endothelial cells. It is defined as myocardial interstitial dilation due to deposition of extracellular matrix (ECM) proteins [1]. Initial deposition of ECM improves tissue integrity, but extensive fibrosis impairs cardiac function [2]. Notably, fibroblasts, particularly myofibroblasts, play a pivotal role as downstream effector cells in the fibrotic process and serve as the primary source of matrix proteins [3]. In addition, ECM metalloproteinases (MMPs) are proteases that specifically regulate the content of ECM. Changes in the frequency and relative proportions of MMPs and their inhibitors can lead to changes in the myocardial matrix [4]. Disruption of this balance is also present in some mechanisms that promote fibrosis, but exactly what is changed remains a matter of debate.

In recent years, an increasing body of literature has provided compelling evidence indicating that OS acts as a mediator in the above pathological state of cells and promotes the progression of cardiac fibrosis. OS is characterized by an imbalance between oxidative and antioxidative processes in the body. The primary cause of OS is the generation of ROS. ROS diffuses within the cytoplasm and activates redox-sensitive protein kinases [5], which play a pro-fibrotic role by promoting fibroblast proliferation, cardiomyocyte apoptosis, and endothelial cell injury or senescence, ultimately leading to extracellular remodeling [6,7]. At the molecular level, OS induces specific manifestations such as cellular electrophysiological disturbances, alterations in the expression of myosin chains [8], oxidative damage to proteins and lipids, DNA damage, and dysregulation of MMPs and their inhibitors [6,9]. Their cumulative effect further leads to the development of cardiac fibrosis, impairing basic heart function. The review aims to provide an extensive overview of the intricate interplay between OS and various cardiac cell types, including cardiomyocytes, cardiac fibroblasts, and endothelial cells. By elucidating the specific epigenetic mechanisms and signaling pathways affected by OS in these cell types, a deeper understanding of the pathogenesis of cardiac fibrosis and remodeling can be gained.

## 2 Epigenetic mechanisms

Epigenetic regulation, a pivotal mediator of fibrotic progression, governs the transcriptional state of genes. It serves as a mechanism for modulating the cellular transcriptome and proteome without affecting the genetic content, potentially accomplished through the establishment of epigenetic marks, including DNA methylation, post-translational modifications of histones, as well as additional mechanisms involving non-coding RNAs [10]. Indeed, numerous studies have proved that OS can alter the epigenetic landscape of cells [11,12]. There even have been reports supporting that superoxides could directly mediate cytosine methylation by deprotonating C5 and transferring the methyl group from SAM without the involvement of DNA methyltransferase [13]. Nonetheless, many studies have also demonstrated the epigenetic mediation of OS [14]. An important example is that activation of eroxisome proliferator-activated receptor gamma coactivator-1 alpha (PGC-1α) reduces mitochondrial ROS. However, the activation of PGC-1α is contingent upon its deacetylation mediated by sirtuins (SIRT) [15]. Overall, redox signaling and OS could modulate gene regulation by altering histone function and DNA-modifying enzymes, which subsequently impact cellular phenotypes. Conversely, epigenetic changes can also influence the redox environment within cells [16].

Meanwhile, epigenetic and OS have important downstream effects, both contributing to fibrosis. It can be seen that OS is closely related to epigenetics, overlaps each other, and significantly affects the progression of cardiac fibrosis. Therefore, modulation of OS through epigenetic mechanisms is a potential and promising therapeutic option for cardiac fibrosis.

## 3 Overview of oxidative stress in cardiac fibrosis

Regulation of redox homeostasis is critical for maintaining normal cell growth, metabolism and survival. OS, characterized by an imbalance of the body's oxidation and antioxidant mechanisms, primarily arises from ROS [17]. ROS is a collective term encompassing molecules derived from O2, such as superoxides, hydrogen peroxide, hydroxyl radicals, ozone, and singlet oxygen [18]. And numerous additional agents also contribute to redox signaling, including nitric oxide, hydrogen sulfide, and oxidized lipids [19]. These agents play an important role in mediating diverse biological processes within the body by modulating protein function, promoting inflammation, inducing apoptosis, impairing autophagy, disrupting mitochondrial function, and interfering with various signaling pathways. These effects tend to accelerate the pathological process and aggravate the disease symptoms.

Intracellular production of ROS can stem from multiple sources, encompassing the mitochondrial electron transport chain (ETC), NADPH oxidase/Dual oxidase (NOX/DUOX) enzymes, endoplasmic reticulum (ER), as well as superoxide generation [20]. The detrimental impacts of ROS are intricately involved in the pathogenesis of cardiac tissue. To counterbalance these effects, the human body possesses a sophisticated antioxidant system consisting of both enzymatic and non-enzymatic components. Among these, crucial elements include antioxidant enzymes, such as superoxide dismutase (SOD), macrophage migration inhibitory factor (MIF), and catalase, alongside various non-enzyme antioxidants. These components collectively contribute to the regulation of OS and maintenance of redox homeostasis in cardiac cells.

**Fig. 1:**
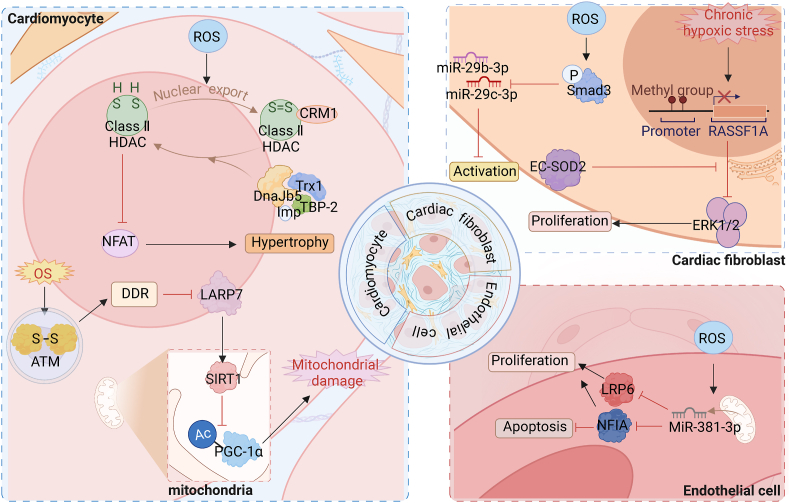
Oxidative stress influences cardiac fibrosis by affecting epigenetic modification ROS affect cardiac fibrosis progression by mediating multiple epigenetic mechanisms in various cardiac tissue cells, including cardiomyocytes, cardiac fibroblasts, and endothelial cells.

Remarkably, several studies have demonstrated that ROS play a significant role in modulating the ECM by enhanced protein expression of TGF-β1, alpha smooth muscle actin, collagen I, and collagen Protein III, leading to the activation of cardiac fibroblasts [21]. TGF-β plays a crucial role in the transdifferentiation of cardiac fibroblasts and the deposition of ECM [22]. Upon activation, fibroblasts exhibit a significant increase in collagen production, while ROS also have the ability to disrupt the balance of MMPs and their inhibitors [9]. These processes collectively contribute to the development of fibrosis and remodeling of the matrix. Moreover, oxidative damage inflicted upon ECM proteins can result in their fragmentation and/or stabilization, consequently impairing the proper resolution of inflammation and fibrosis. In addition, ROS are also involved in signaling, such as apoptosis signaling [23]. Apoptosis is usually associated with the activation of caspase proteases. Apoptosis stimulated by ROS may also aggravate cardiac fibrosis [24]. We recently discovered that these ROS-induced changes are inextricably linked to concomitant epigenetic changes. Below we discuss recent studies of OS-regulated epigenetic changes mediating myocardial fibrosis, Fig. 1 and Table 1 provide more details on this discovery.

**Table 1:** Oxidative stress influences cardiac fibrosis by affecting epigenetic modification.

	DNA methylation	Histone modification	Non-coding RNA	
Transformation	●DNA methylation ↑ inactivate RASSF1A activate ERK1/2 [27] Proliferation	●activate DDR signaling pathway SIRT1↓ PGC-1α acetylation [29] Mitochondrial damage ●HDAC4 is transported to the cytoplasm [30] Hypertrophy	●Activate Smad3 miR-29b-3p和miR-29c-3p↓ target TGF-β2 and MMP2 [32] Activation	●redistribution of miR-381–3p between mitochondria and cytoplasm inhibiting LRP6 and NFIA [33] Apoptosis Inhibit proliferation
Cell type	Cardiac fibroblast	Cardiomyocyte	Cardiac fibroblast	Endothelial cell

## 4 Oxidative stress mediated epigenetic regulations of cardiac fibrosis

OS exerts a substantial influence on the induction of epigenetic modifications, with a particular focus on DNA methylation. Adverse effects of oxidative damage on DNA integrity disrupt the chromatin structure and lead to epigenetic changes, leading to subsequent epigenetic alterations. This interplay between OS and epigenetic modifications highlights the intricate relationship between environmental cues and the modulation of gene expression patterns [25]. ROS have the capacity to modify the methylation status of CpG sites, impeding their interaction with transcription factors that would typically bind to them. Furthermore, studies have provided evidence indicating that OS transiently influences epigenetic processes by modulating the activity of enzymes involved in histone demethylation and deacetylation [26].

### 4.1 Antioxidants reduce DNA methylation

RASSF1A is a tumor suppressor gene that exerts its effects by modulating downstream proteins, including ERK1/2. The Ras/ERK signaling pathway represents an intracellular signaling cascade intricately associated with fibroblast proliferation. Notably, DNA methylation events occurring in cardiac fibroblasts lead to the inactivation of the tumor suppressor gene RASSF1A, concurrently activating ERK1/2 and culminating in fibroblast proliferation and the development of cardiac fibrosis. Chronic exposure to hypoxic stress triggers DNA methylation events, while extracellular superoxide dismutase (EC-SOD) exhibits a significant capacity to mitigate RASSF1A gene methylation and alleviate hypoxia-induced cardiac fibrosis [27].

### 4.2 Oxidative stress affects histone modification

Previous investigations have provided evidence indicating that the activation of ROS-induced ataxia-telangiectasia mutated (ATM) protein kinase is evident in conditions of hypertrophy and heart failure [28]. In cardiomyocytes, OS triggers the activation of the ATM-mediated DNA damage response (DDR) signaling pathway, subsequently leading to the degradation of La-related protein 7 (LARP7). The downregulation or mutation of LARP7 elicits a reduction in SIRT1 expression, resulting in the acetylation of PGC-1α and impairing mitochondrial biogenesis and energy metabolism. Ultimately, these cascades contribute to the progression of heart failure, with cardiac fibrosis emerging as a critical pathological manifestation [29]. Adeno-associated virus (AAV)-mediated delivery of LARP7 emerges as a promising targeted therapeutic approach for addressing cardiac dysfunction.

Alterations in the redox status of conserved cysteine residues within class II histone deacetylases (HDACs) exert a significant impact on the cytoplasmic localization of these HDACs and the activity of crucial genes implicated in cardiomyocyte hypertrophy. These cysteine mutations are sufficient to induce cardiac hypertrophy [30]. Cardiomyocyte hypertrophy is a prominent feature of cardiac hypertrophy, which can be categorized into pathological hypertrophy and physiological hypertrophy. Pathological hypertrophy is triggered by disease stimuli such as stress overload or myocardial infarction, and myocardial fibrosis serves as a notable manifestation of pathological hypertrophy [31]. When exposed to OS, HDAC4 mutants rely on chromosome region maintenance protein 1 (CRM1) for their translocation into the cytoplasm, thereby abolishing the inhibitory effect of HDAC4 on cardiac hypertrophy in vivo. Interestingly, thioredoxin 1 (TRX1) plays a crucial role in monitoring and regulating redox signaling [16]. It forms a multiprotein complex with DnaJb5, thioredoxin-binding protein-2 (TBP-2), and importin (IMP) α, facilitating the degradation of key cysteine residues within HDAC4 and promoting the nuclear localization of HDAC4. Consequently, targeted redox modulation of HDAC4 represents a promising independent approach for the therapeutic management of cardiac hypertrophy.

### 4.3 Oxidative stress affects the regulation of non-coding RNAs

Moreover, increased levels of ROS have been shown to downregulate the expression of miR-29b-3p and miR-29c-3p by activating Smad3 signaling. This dysregulation of microRNAs contributes to the promotion of cardiac fibrosis in cardiac fibroblasts (CFs) by targeting TGF-β2 and MMP2 [32]. Notably, macrophage migration inhibitory factor (MIF) possesses antioxidant properties and has been found to inhibit ROS production and inhibit the activation of Smad3. As a result, MIF intervention restores the downregulation of miR-29b-3p and miR-29c-3p in CFs, thereby exerting a suppressive effect on cardiac fibrosis.

Another ROS-mediated mechanism involving microRNAs (miRNAs) has been identified. It is characterized by the ROS-induced redistribution of miRNAs between mitochondria and the cytoplasm, specifically in endothelial cells, leading to endothelial cell damage. Importantly, this redistribution does not significantly alter the overall expression of miRNAs in the cell. Among these redox-sensitive miRNAs, miR-381–3p has been implicated in ROS-induced endothelial injury through this mechanism. It exerts its effects by targeting specific genes, including lipoprotein receptor-related protein 6 (LRP6) and nuclear factor I-A (NFIA). By inhibiting these target genes, miR-381–3p promotes apoptosis and inhibits endothelial cell proliferation, contributing to the progression of endothelial dysfunction [33].

## 5 Epigenetic regulations of oxidative stress in cardiac fibrosis: focus on histone modification

**Fig. 2:**
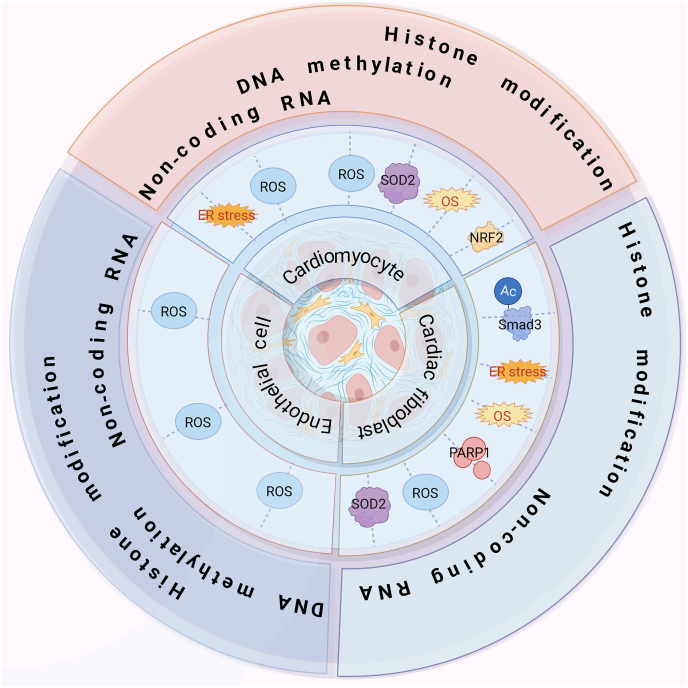
Epigenetic modification influences cardiac fibrosis by affecting oxidative stress Several epigenetic mechanisms influence cardiac fibrosis progression by affecting oxidative homeostasis in vivo.

As previously mentioned, OS has been shown to induce alterations in the epigenetic landscape of cells. In the following sections, we discuss the epigenetic mechanisms that mediate OS. Additional insights and detailed information can be found in Fig. 2 and Table 2, which provide a comprehensive overview of these discoveries.

**Table 2:** Epigenetic modification influences cardiac fibrosis by affecting oxidative stress.

	Cardiac fibroblast	Cardiomyocyte	Endothelial cell
DNA methylation	−/−	●DNMT2 decreases GPX1 expression [54] Apoptosis↑	●DNA methylation levels of the IGFBP3 promoter↑ nuclear translocation of IGFBP3 [55] Angiogenesis↑
Histone modification	●SIRT1 inhibits Smad3 acetylation OS and ER stress [39] Activation↓	●SIRT1 reduces nuclear translocation of NRF2 [40] Ferroptosis↑ ●SIRT3 induces IDH2 deacetylation [49] Apoptosis↓ ●SIRT3 increase SOD2 activity [47] Apoptosis↓ ●SIRT4 inhibits Sirt3-mediated activation of MnSOD [50] Hypertrophy↑ ●EZH2 inhibits the expression of USP22 [52] Apoptosis↑	●SIRT1 inhibits AngII-induced ROS generation [43] Aging↑
Non-coding RNA	●miR-4732–3p increases Nfe2l2 and NRF1 [63] Activation↓	●miR-134–5p inhibits the expression of KAT7 [61] Hypertrophy↑ ●miR-194–5p Inhibits PAK2 expression [62] Apoptosis↑ ●miR-340–5p inhibits the expression of Mcl-1 [63] Apoptosis↑ ●lncDACH1 promote SIRT3 ubiquitination [65] Apoptosis↑ ●CircSamd4 induces the mitochondrial translocation of the Vcp protein [66] Apoptosis↓ ●CircITCH Upregulates SIRT6 [67] Apoptosis↓	miR-101–3p inhibits expression of Bim [59] Apoptosis↓

**Fig. 3:**
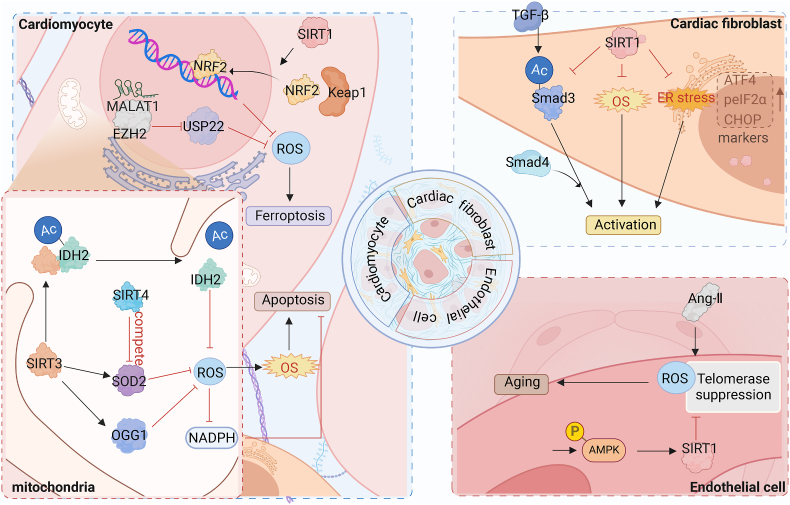
Regulatory effect of histone modification on oxidative stress in cardiac fibrosis Histone modifications affect the intracellular oxidative balance of various cardiac tissue cells such as cardiomyocytes, cardiac fibroblasts, and endothelial cells, thereby affecting the process of cardiac fibrosis. Among them, ATF4, peIF2α and CHOP were upregulated as markers of ER stress.

One prominent epigenetic mechanism that has been extensively studied is histone modification. Fig. 3 illustrates the intricate regulation of OS by histone modifications. Among various histone modifications, acetylation and methylation have garnered significant attention due to their roles in modulating chromatin condensation, transcription factor binding, and transcription elongation [34]. These modifications are meticulously controlled and manipulated by three classes of proteins, often referred to as “writers” “erasers” or “readers” based on their specific functions [35].

“Writers” encompass enzymes such as HATs and HMTs, which catalyze the addition of acetyl and methyl groups to histones, respectively. On the other hand, “erasers” include proteins like deacetylases and demethylases, which are responsible for removing acetyl and methyl groups from histones. Lastly, “readers” refer to proteins that bind to these modified histone marks, exerting their regulatory influence on chromatin organization and the activity of other proteins.

The intricate interplay between OS and histone modifications reveals the dynamic nature of epigenetic regulation in response to the cellular redox status. These complex mechanisms are pivotal in shaping the epigenome, exerting profound effects on gene expression, cellular functionality, and the pathogenesis of various diseases. Elucidating the intricate network of interactions between OS and histone modifications holds significant promise for advancing our understanding of epigenetic regulation and its therapeutic implications in diverse pathological contexts.

### 5.1 Histone deacetylation regulations of oxidative stress in cardiac fibrosis

Mitochondria plays a vital role in generating ROS within cells [36]. In recent years, the epigenetic mechanisms associated with SIRTs, particularly those involving mitochondrial proteins, have garnered considerable research interest. SIRTs are enzymes with nicotinamide adenine dinucleotide (NAD+)-dependent activity, and their activation is intricately regulated by the metabolic state of the cell.

Activation of SIRT1, for instance, leads to an elevation in adiponectin levels, which in turn stimulates mitochondrial biogenesis and enhances the antioxidant defense system. SIRT1 interacts with peroxisome PGC-1α at multiple lysine and deacetylation sites, resulting in increased transcription of genes involved in oxidative phosphorylation [37]. In response to stressful conditions, SIRT1 exerts its regulatory function by deacetylating forkhead box protein O1/3 (FOXO1/3), thereby regulating the expression of manganese-dependent superoxide dismutase and catalase antioxidants [38]. Furthermore, Activated SIRT1 inhibits Smad3 acetylation, OS, and activation of ER stress, thereby preventing the activation of cardiac fibroblasts. Geniposide (GE) can resist cardiac fibrosis through this mechanism, so it is expected to become a promising anti-cardiac fibrosis drug [39].

Moreover, Impairment or loss of cardiac SIRT1 contributes to the exacerbation of cardiac dysfunction and remodeling. Inhibition of SIRT1 reduces the nuclear translocation of NRF2, leading to a decrease in NRF2 translocation from the cytoplasm to the nucleus. This alteration affects the downstream Keap1 pathway, compromising the ability of NRF2 to effectively inhibit ROS in cardiomyocytes [40]. NRF2 primarily functions to maintain redox homeostasis [16] by transcriptionally regulating various genes involved in antioxidant defense, such as SOD, catalase (CAT), heme oxygenase 1 (Hmox-1), NAD(P)H quinone oxidoreductase-1, and TRX-1 [15]. Consequently, the levels of MDA, a marker of OS, increase, while the levels of SOD and GSH, key antioxidants, decrease. And honokiol (HKL), a potent cardioprotectant, exerts its beneficial effects by stimulating the SIRT1/NRF2 pathway, thereby reducing OS and ameliorating myocardial injury in diabetic rats [41]. Moreover, HKL also serves as a pharmacological activator of SIRT3 [42].

Activation of SIRT1 plays a crucial role in inhibiting angiotensin II (AngII)-induced ROS generation and promoting telomerase activity, thereby attenuating AngII-induced senescence in human umbilical vein endothelial cells (HUVECs) [43]. Notably, apelin, an adipocyte-derived factor, acts as an endogenous ligand and the activation of its receptors has been shown to ameliorate the endothelia in-induced HUVEC senescence through the AMPK/SIRT1 signaling pathway.

Previous studies have shown that moderate expression of SIRT1 confers protection against OS associated with cardiac hypertrophy and fibrosis. However, it is important to note that higher levels of SIRT1 expression may actually contribute to cardiomyopathy by promoting mitochondrial dysfunction [44]. Therefore, while activated SIRT1 holds promise as a potential therapy for cardiac fibrosis, careful consideration of dosage is necessary to achieve the desired therapeutic effects without causing adverse effects on mitochondrial function.

SIRT3 plays a crucial role as the major mitochondrial SIRT in regulating 8-oxoguanine DNA glycosylase (OGG1) and mitochondrial manganese superoxide dismutase [45]. Deficiency of SIRT3 leads to the downregulation of several mitochondrial proteins, including Mn-SOD and OGG1, resulting in elevated mitochondrial ROS levels and mitochondrial DNA (mtDNA) damage. A study has confirmed that SIRT3 is the predominant protein deacetylase in mitochondria, and its loss results in a nearly tenfold increase in acetylation modifications of mitochondrial proteins [46]. Loss of SIRT3 leads to hyperacetylation of the key mitochondrial antioxidant SOD2, which induces OS and promotes apoptosis in cardiomyocytes, leading to cardiac fibrosis. Neuraminidase 1 (NEU1) can exploit this mechanism to control OS, thereby reducing cardiac fibrosis and is therefore a potential therapeutic target [47]. As SIRT3 expression increased, SOD2 deacetylation decreased and SOD2 activity increased, limiting ROS accumulation. In addition, SIRT3 also regulates the expression of NAPDH oxidase through mitochondrial ROS [48].

Transient stimulation of nonischemic hypertrophy has been shown to enhance the cardiac resilience to subsequent ischemic stress, and this process is partly mediated by SIRT3. SIRT3 plays a crucial role by directly binding to and deacetylating Isocitrate dehydrogenase 2 (IDH2), resulting in increased IDH2 activity. The activation of IDH2 could inhibit mitochondrial ROS production and mitigates mitochondria-dependent apoptosis through the production of mitochondrial nicotinamide adenine dinucleotide phosphate (NADPH) [49].

However, emerging evidence suggests that SIRT4 plays a contrasting role. In cardiomyocytes, SIRT4 inhibits SIRT3-mediated activation of Mn-SOD, resulting in increased ROS levels that contribute to the development of pathological cardiac hypertrophy. This mechanism may involve a competitive interaction between SIRT4 and Mn-SOD for binding to SIRT3 [50]. Therefore, targeting the inhibition of SIRT4-mediated OS presents a potential therapeutic strategy for mitigating pathological hypertrophy and managing heart failure. Further studies are warranted to elucidate the precise molecular mechanisms underlying the role of SIRT4 in cardiac pathophysiology.

Additionally, lisinopril, a novel non-sulfhydryl angiotensin-converting enzyme (ACE) inhibitor, has been shown to enhance the antioxidant defense system in animals and humans, offering protection against oxidative damage and fibrosis in human cardiomyocytes through epigenetic mechanisms [51]. Following treatment with lisinopril, activation of SIRT1 and SIRT6 signaling pathways has been observed, imparting a protective effect against OS and fibrosis in human AC16 cardiomyocytes. Notably, the expression of antioxidative stress proteins such as catalase, SOD2, and TRX was significantly increased in cardiomyocytes, while key proteins implicated in cardiac fibrosis, namely osteopontin and Galectin-3, were significantly decreased.

SIRTs have emerged as crucial regulators of OS and have garnered significant attention in the study of metabolic disorders due to their involvement in epigenetic modifications within mitochondria. Further investigations into the intricate interplay between SIRTs, OS, and epigenetic regulation offer considerable potential for advancing our knowledge of cardiac fibrosis diseases and uncovering novel therapeutic strategies.

### 5.2 Histone methylation regulations of oxidative stress in cardiac fibrosis

Metastasis-associated lung adenocarcinoma transcript 1 (MALAT1), a lncRNA, has emerged as a crucial regulator of apoptosis in diabetic cardiomyopathy through its interaction with enhancer of EZH2. MALAT1 facilitates the recruitment of EZH2 to the myocardial nucleus, thereby promoting EZH2 activity. Consequently, EZH2 suppresses the expression of ubiquitin-specific peptidase 22 (USP22) by modifying H3K27me3, leading to enhanced OS, inflammation, and apoptosis in rat cardiomyocytes [52]. Moreover, MALAT1 has been observed to interact with EZH2 in cardiac microvascular endothelial cells. Genetic and biochemical studies have demonstrated the regulatory role of histone methylation in DNA methylation, while histone acetylation influences histone methylation dynamics. These intricate epigenetic modifications collectively contribute to the complex regulatory landscape underlying cardiomyopathy [53].

## 6 Epigenetic regulations of oxidative stress in cardiac fibrosis: focus on DNA methylation

**Fig. 4:**
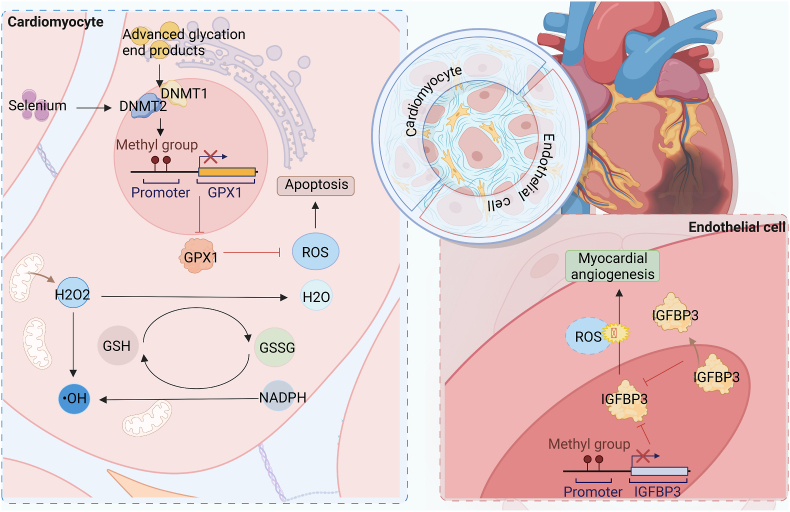
Regulatory effect of DNA methylation on oxidative stress in cardiac fibrosis In cardiomyocytes and endothelial cells, DNA methylation mediates the expression of key proteins that regulate OS, thereby affecting cellular oxidative balance and consequently cardiac fibrosis.

DNA methylation, as depicted in Fig. 4, is a crucial epigenetic mechanism involved in the modulation of cardiac fibrosis under conditions of OS. It is the earliest and extensively studied epigenetic modification, exerting regulatory control over various cellular processes such as transcriptional regulation, transposon silencing, maintenance of genomic imprinting, and X chromosome inactivation. Methylation-induced gene silencing entails a complex interplay of DNA-protein and protein-protein interactions, along with a cascade of enzymatic activities responsible for dynamic changes in DNA methylation patterns. Key players in this process include DNMTs and TET enzymes, which collectively orchestrate the delicate balance of DNA methylation dynamics. Understanding the intricate mechanisms underlying DNA methylation-mediated gene regulation holds immense potential in unraveling the pathogenesis of cardiac fibrosis and may pave the way for the development of targeted interventions.

Within cardiomyocytes, DNMT2 has been observed to induce DNA methylation within the promoter region of the glutathione peroxidase 1 (GPX1) gene, consequently diminishing the expression of GPX1. As a representative selenoprotein, GPX1 plays a key role in reducing organic hydrogen peroxide to water or alcohols utilizing GSH as a reducing agent. Insufficient GPX1 levels lead to compromised antioxidant defense, cellular dysfunction, and apoptosis [54], thereby facilitating the progression of fibrosis. Consequently, selenium supplementation emerges as a potential therapeutic strategy to ameliorate the levels of ROS and enhance cardiac function. By replenishing selenium levels, it is hypothesized that ROS generation can be reduced, culminating in an improvement in overall cardiac health. Further investigations in this domain hold promise for the development of targeted therapeutic interventions aimed at mitigating OS-related pathologies in the heart.

Salvianolic acid B (Sal B) has been demonstrated to exert beneficial effects on diabetic cardiomyopathy (DCM) by promoting angiogenesis, as well as attenuating cardiac fibrosis and remodeling through the inhibition of IGFBP3 [55]. The underlying molecular mechanism involves Sal B's ability to enhance DNA methylation within the promoter region of IGFBP3 under hypoxic conditions. This modification leads to the translocation of IGFBP3 from the nucleus to the cytoplasm, subsequently resulting in its downregulation. As a consequence, cardiac angiogenesis is promoted, cardiac fibrosis is reduced, and overall heart function is improved [56]. Previous studies have indicated that IGFBP3 is implicated in OS regulation and apoptosis in various tissues [57,58], yet its involvement in cardiac fibrosis has remained unclear. Further investigations are warranted to elucidate the precise role of IGFBP3 in the pathogenesis of cardiac fibrosis and to explore its potential as a therapeutic target for this condition.

## 7 Epigenetic regulations of oxidative stress in cardiac fibrosis: focus on non-coding RNAs

**Fig. 5:**
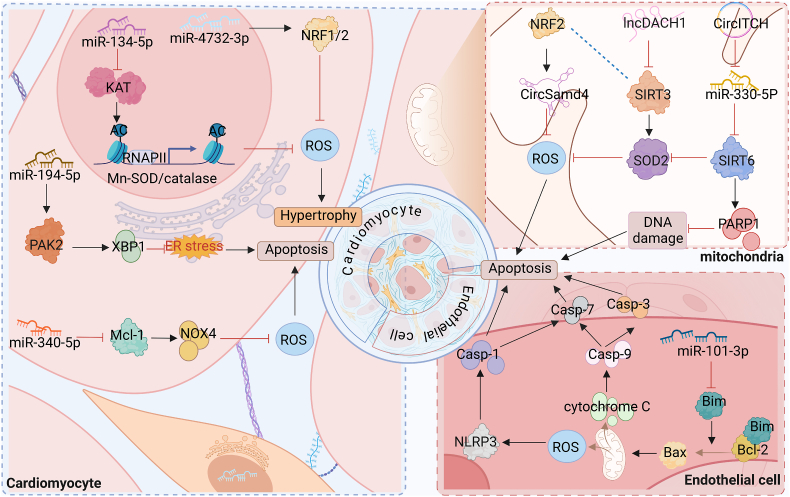
Regulatory effect of non-coding RNA on oxidative stress in cardiac fibrosis In cardiomyocytes and endothelial cells, non-coding RNAs mediate the expression of key proteins that regulate OS, thereby affecting cellular oxidative balance and consequently cardiac fibrosis.

Non-coding RNAs (ncRNAs) consist of a variety of RNA molecules not normally involved in protein coding. This group encompasses various types of ncRNAs, including microRNAs, siRNAs, lncRNAs, and circRNAs. Importantly, these ncRNAs play pivotal roles in mediating genetic changes. By exerting regulatory functions at the epigenetic levels, ncRNAs contribute to the intricate molecular mechanisms underlying the interplay between OS and the development of cardiac fibrosis, as depicted in Fig. 5.

### 7.1 MiRNA regulations of oxidative stress in cardiac fibrosis

miRNAs have emerged as promising targets and regulators of OS-related signaling pathways, exerting their effects by binding to the 3′-UTR of target mRNAs. Notably, various miRNAs have been implicated in the regulation of ROS production in the heart. Among these miRNAs are miR-21, miR-30, miR-34a, miR-144, miR-140–5p, miR-181, miR-378, miR-421, and miR-451. These miRNAs play crucial roles in modulating ROS levels by directly or indirectly targeting genes involved in ROS generation, detoxification, or antioxidant defense mechanisms.

Moreover, a decrease in miR-101–3p levels in endothelial cells has been associated with an upregulation of Bim expression, an important initiator of the intrinsic apoptosis pathway. Bim interacts with B-cell lymphoma-2 (Bcl-2), leading to the release of Bcl-2-associated X (Bax) and subsequent promotion of ROS production. Endothelial cell apoptosis is then induced by NLRP3 inflammasome-mediated activation of caspase-1/7 [59]. On the contrary, another study demonstrated that miR-101 can indirectly enhance Bim expression through EZH2-dependent epigenetic regulation, thereby sensitizing tumor cells to apoptosis induced by chemotherapeutic drugs [60]. These observations highlight the role of miR-101 in modulating apoptotic pathways and provide insights into potential therapeutic strategies targeting miR-101 and its downstream effectors to regulate apoptosis and ROS-related processes in various pathological contexts.

miR-134–5p has been found to be highly expressed in a mouse model of myocardial infarction. Recent studies have elucidated that miR-134–5p plays a role in regulating ROS levels through its interaction with lysine acetyltransferase 7 (KAT7), a HAT responsible for acetylating histone H3 at lysine 14 (H3K14Ac) in Mn-SOD and catalase genes. Mechanistically, miR-134–5p suppresses the expression of KAT7, leading to reduced levels of Mn-SOD and catalase, which in turn promotes ROS accumulation. This dysregulation of ROS homeostasis ultimately contributes to the activation of cardiac fibroblasts, processes associated with cardiac fibrosis and adverse remodeling [61].

Inhibition of miR-194–5p has been demonstrated to mitigate doxorubicin (Dox)-induced cardiomyocyte apoptosis. Specifically, miR-194–5p directly targets and suppresses the expression of p21-activated kinase 2 (PAK2). PAK2 has been previously reported to exert cardioprotective effects by enhancing ER function through the activation of the IRE1/XBP1 signaling pathway. Activation of XBP1, in turn, leads to upregulation of ER chaperones, alleviation of ER stress, and promotion of cell survival. Upon treatment with Dox, there is a dynamic alteration in the expression of XBP1s, with an initial increase followed by a subsequent decrease. Notably, inhibition of miR-194–5p results in the overexpression of XBP1, thereby restoring its levels even after Dox treatment. Consequently, this leads to the inhibition of dox-induced caspase 3/7 activity and an increased expression of cleaved caspase 12, ultimately culminating in the protection of cardiomyocytes from apoptotic cell death [62].

miR-340–5p has been identified as a suppressor of myeloid cell leukemia 1 (Mcl-1) expression. Notably, Mcl-1 possesses a unique ability to impede ROS formation by inhibiting the upregulation of pro-oxidants through NOX4. Consequently, overexpression of miR-340–5p exacerbates mitochondrial dysfunction and increases OS, ultimately leading to enhanced apoptosis. Furthermore, miR-340–5p also modulates the expression levels of key apoptotic regulators, including Bim, Bax, and cleaved caspase 3, while reducing the expression of the anti-apoptotic protein Bcl-2. These molecular changes collectively contribute to the protection of cardiomyocytes from apoptosis [63].

MiR-4732–3p has emerged as a potential mediator of cardioprotective mechanisms in rat cardiac cells and cardiac fibroblasts, primarily through its ability to enhance antioxidant responses. Notably, miR-4732–3p has been shown to upregulate the mRNA levels of two critical transcription factors, Nfe2l2 and NRF1. These transcription factors are known to regulate genes encoding proteins involved in the cellular response to free radical damage [64].

### 7.2 LncRNA regulations of oxidative stress in cardiac fibrosis

In the context of DCM, recent investigations have revealed elevated expression of the long non-coding RNA lncDACH1 in DCM hearts and cardiomyocytes exposed to high glucose levels. Notably, knockdown of lncDACH1 has demonstrated favorable effects, including the reduction of mitochondrial OS, apoptosis, cardiac fibrosis, and hypertrophy, ultimately improving heart function in DCM mice.

Further studies have shed light on the underlying mechanisms by which lncDACH1 exerts its effects. It has been discovered that lncDACH1 directly interacts with SIRT3. This interaction facilitates the binding of SIRT3 to E3 ligases or other components of the ubiquitin-proteasome system, thereby promoting the ubiquitination process. This ubiquitination leads to the degradation of SIRT3, resulting in decreased activity of Mn-SOD, a crucial antioxidant enzyme. As a consequence, the imbalanced redox state leads to increased levels of ROS, ultimately causing cellular injury and potentially triggering apoptosis in mouse cardiomyocytes [65].

### 7.3 CircRNA regulations of oxidative stress in cardiac fibrosis

CircRNAs localized within mitochondria have emerged as critical regulators of mitochondrial ROS production. Among these, circSamd4 is under the transcriptional control of the NRF2 transcription factor, which binds to the promoter region of the circSamd4 host gene. Functionally, circSamd4 mitigates OS by facilitating the translocation of valosin-containing proteins (VCPs) to the mitochondria, thereby maintaining mitochondrial dynamics. This process leads to a reduction in the expression of voltage-dependent anion channel 1 (VDAC1) and blocks the opening of the mitochondrial permeability transition pore (mPTP). Consequently, mitochondrial OS and subsequent oxidative DNA damage are alleviated, inducing cardiomyocyte (CM) proliferation and preventing CM cell apoptosis when CircSamd4 was overexpressed, and the area of fibrosis was also smaller. Conversely, silencing circSamd4 has the opposite effect, exacerbating OS, impairing mitochondrial function, and promoting adverse cardiac remodeling [66].

Moreover, circular RNA itchy E3 ubiquitin protein ligase (CircITCH), a tumor suppressor with broad-spectrum functionality, has emerged as a key player in doxorubicin-induced cardiotoxicity. The protein ITCH is an important enzyme involved in the transfer of ubiquitin from E2 ubiquitin-conjugating enzymes to specific protein substrates, thereby marking them for lysosomal degradation. CircITCH upregulates SIRT6 by sponge filtering mmir-330–5p in the mouse heart [67]. SIRT6, in turn, mitigates OS by activating NRF2 [68] and Mn-SOD2, both of which are crucial endogenous defense molecules against oxidative damage. Additionally, SIRT6 enhances DNA damage repair by activating PARP1, a pivotal enzyme involved in DNA repair mechanisms [69]. Collectively, these molecular events culminate in improved cardiomyocyte survival and enhanced cellular resilience in the face of doxorubicin-induced cardiotoxicity.

## 8 Conclusions and future perspective

Cardiac fibrosis is a prevalent pathological complication of various heart diseases, including cardiac hypertrophy, diabetic cardiomyopathy, coronary heart disease (CHD), hypertensive heart disease, and heart failure. Different types of myocardial fibrosis, such as interstitial, periarteriolar, or “replacement” fibrosis, are associated with distinct underlying pathophysiological processes, which poses challenges in developing therapeutics for myocardial fibrosis [31]. Cardiac hypertrophy is characterized by the prominent presence of cardiac interstitial fibrosis. On the other hand, diabetic cardiomyopathy is characterized by diffuse myocardial fibrosis (interstitial fibrosis at the microscopic level), along with the occurrence of endothelial-to-mesenchymal transition (EndMT) and cardiac hypertrophy [70]. In the context of CHD, studies often investigate models of myocardial infarction and myocardial ischemia-reperfusion, where OS-induced cardiomyocyte apoptosis plays a significant role in disease progression. Replacement or scar fibrosis, which involves the formation of fibrotic tissue to replace damaged or necrotic cardiomyocytes [71], is a common occurrence in CHD models. In hypertension, fibrosis is initiated in the perivascular space [72], and it has been demonstrated that hypertensive heart disease is characterized by predominant perivascular fibrosis in both human and murine models [73].

Cardiac fibrosis will inflict significant damage, and currently lacks effective preventive or reversal treatments. ROS play a crucial role in the pathogenesis of cardiac fibrosis, orchestrating several pathological changes such as cardiomyocyte apoptosis, heightened activation of cardiac fibroblasts, and increased senescence and damage in endothelial cells, collectively contributing to fibrosis progression. Recent studies have increasingly implicated OS and its interplay with epigenetic modifications in the context of cardiac fibrosis. This comprehensive review summarizes the latest research in this area and proposes that OS may influence cardiac fibrosis through its modulation of epigenetic mechanisms, while epigenetic modifications may, in turn, ameliorate cardiac fibrosis by attenuating OS. Nevertheless, the precise underlying mechanisms are not yet fully elucidated, and given the overlap of various epigenetic pathways, further investigations are warranted to determine the impact of epigenetic-mediated OS on the fibrotic process.

Furthermore, it is important to acknowledge the signaling function of ROS, as they serve as essential mediators of cellular signaling and regulation. Thus, it is crucial to recognize the multifaceted role of ROS when designing therapeutic interventions. In conclusion, targeting epigenetic OS for the treatment of fibrosis shows promise as a therapeutic direction. However, there is a need to explore other epigenetic mechanisms involved in the regulation of OS, as well as additional bioactive substances governed by epigenetic modifications. Further experimental and clinical studies are essential to ascertain the potential of epigenetic-based therapeutic strategies in addressing OS during the progression of cardiac fibrosis.

## Author contributions

All authors conceived the manuscript structure and contributed to the writing and editing.

## Funding

This project was supported by 10.13039/501100001809National Natural Science Foundation of China (82170236, 81700212), 10.13039/501100017668Key research and development projects of Anhui Province (202104j07020037), Translational medicine research project of Anhui Province (2021zhyx-C61), Excellent Top Talents Program of Anhui Province Universities (gxyqZD2022023), and 10.13039/501100001809National Natural Science Foundation Incubation Program of the Second Affiliated Hospital of 10.13039/501100002947Anhui Medical University (2020GMFY02). Postgraduate Innovation Research and Practice Program of 10.13039/501100002947Anhui Medical University (YJS20230083, YSJ20230082).

## Declaration of competing interest

The authors declare that they have no known competing financial interests or personal relationships that could have appeared to influence the work reported in this paper.

## Data Availability

Data will be made available on request.
